# Heat-Shock Induces Granule Cell Dispersion and Microgliosis in Hippocampal Slice Cultures

**DOI:** 10.3389/fcell.2021.626704

**Published:** 2021-02-22

**Authors:** Jasmin Weninger, Maurice Meseke, Shaleen Rana, Eckart Förster

**Affiliations:** Institute of Anatomy, Department of Neuroanatomy and Molecular Brain Research, Ruhr-Universität Bochum, Bochum, Germany

**Keywords:** epilepsy, Cajal–Retzius cells, Reelin, CNS inflammation, hippocampus, Prox-1

## Abstract

Granule cell dispersion (GCD) has been found in the dentate gyrus (dg) of patients with temporal lobe epilepsy (TLE) and a history of febrile seizures but was also recently observed in pediatric patients that did not suffer from epilepsy. This indicates that GCD might not always be disease related, but instead could reflect normal morphological variation. Thus, distribution of newborn granule cells within the hilar region is part of normal dg development at early stages but could be misinterpreted as pathological GCD. In turn, pathological GCD may be caused, for example, by genetic mutations, such as the reeler mutation. GCD in the reeler mutant goes along with an increased susceptibility to epileptiform activity. Pathological GCD in combination with epilepsy is caused by experimental administration of the glutamate receptor agonist kainic acid in rodents. In consequence, the interpretation of GCD and the role of febrile seizures remain controversial. Here, we asked whether febrile temperatures alone might be sufficient to trigger GCD and used hippocampal slice cultures as *in vitro* model to analyze the effect of a transient temperature increase on the dg morphology. We found that a heat-shock of 41°C for 6 h was sufficient to induce GCD and degeneration of a fraction of granule cells. Both of these factors, broadening of the granule cell layer (gcl) and increased neuronal cell death within the gcl, contributed to the development of a significantly reduced packaging density of granule cells. In contrast, Reelin expressing Cajal–Retzius (CR) cells in the molecular layer were heat-shock resistant. Thus, their number was not reduced, and we did not detect degenerating CR cells after heat-shock, implying that GCD was not caused by the loss of CR cells. Importantly, the heat-shock-induced deterioration of dg morphology was accompanied by a massive microgliosis, reflecting a robust heat-shock-induced immune response. In contrast, in the study that reported on GCD as a non-specific finding in pediatric patients, no microglia reaction was observed. Thus, our findings underpin the importance of microglia as a marker to distinguish pathological GCD from normal morphological variation.

## Introduction

Epilepsy encompasses a variety of neurological pathologies, in particular recurrent seizures of hyperexcitable nerve cells and the associated imbalance between excitation and inhibition (Bozzi et al., 2012). Although knowledge on the various causes of these paroxysmal synchronous neuronal discharges in the brain has significantly increased in recent years, the underlying pathological mechanisms, including immunological responses to epileptic seizures, are far from being understood. Complex febrile seizures in early life are thought to be associated with the development of temporal lobe epilepsy (TLE), a disease with strong clinical relevance, and are also suspected to be causally linked to hippocampal lesions later in life (Rocca et al., 1987; Abou-Khalil et al., 1993; Cendes et al., 1993; French et al., 1993; Bender et al., 2004; Vezzani and Granata, 2005; Dube et al., 2009; Koyama, 2013). Juvenile complex febrile seizures were reported to induce dentate granule cell ectopia (Koyama et al., 2012), possibly caused by impaired Reelin signaling. Moreover, granule cell dispersion (GCD) is frequently observed in hippocampal tissue affected by TLE (Houser, 1990; Lurton et al., 1997; Haas et al., 2002; Gong et al., 2007; Blumcke et al., 2009; Haas and Frotscher, 2010), and previous studies have shown that granule cell displacement can lead to incorrect connections of these neurons, which may favor the development of epileptic discharges (Dashtipour et al., 2001; Kowalski et al., 2010; Cameron et al., 2011). In turn, GCD has also been observed in the dentate gyrus (dg) of pediatric patients that did not suffer from epilepsy, suggesting that GCD may be within the normal variation range of granule cell layer (gcl) morphology (Roy et al., 2020).

The extracellular matrix protein Reelin controls several aspects of cortical development and function, in particular the positioning of radially migrating neurons and layer formation, including formation of the dentate gcl (Frotscher, 1998; Rice and Curran, 2001; Tissir and Goffinet, 2003; Forster et al., 2006b; Folsom and Fatemi, 2013). In the reeler mutant mouse, which does not express Reelin, cortical neurons display characteristic migration defects. In the reeler hippocampus, dentate granule cells are not organized in a compact layer but dispersed throughout the hilar region, reminiscent of pathological GCD (D’Arcangelo et al., 1995; Forster et al., 2006b). Therefore, loss of the extracellular matrix protein Reelin, expressed and secreted by CR cells in the dentate molecular layer, is suspected to be a possible cause for the development of GCD in the epileptic hippocampus (Haas et al., 2002; Heinrich et al., 2006; Muller et al., 2009; Duveau et al., 2011).

An increasing number of different clinical and experimental studies point to a correlation between the pathophysiology of epilepsy and inflammation in the central nervous system, including the accumulation, proliferation, and activation of glial cells, in particular of microglia (Choi and Koh, 2008; Vezzani and Ruegg, 2011; Vezzani et al., 2011). Microglia activity promotes cellular immunity and restores physiological homeostasis in the brain by phagocytosis of cell debris and pathogens (Hanisch and Kettenmann, 2007; Devinsky et al., 2013). However, little is known about the interplay between cells of the immune system and neurons in the central nervous system during heat-shock and the subsequent degenerative and regenerative processes.

Organotypic slice cultures of the hippocampal region are a well-established *in vitro* model to study development, function, and plasticity of hippocampal neurons and glial cells in health and disease in a tissue context that is similar to the *in vivo* situation (Forster et al., 1993; Del Rio et al., 1996; Forster et al., 2006b; del Rio and Soriano, 2010; Koyama, 2013). To study the impact of a transient temperature increase on the dg, the incubation temperature of hippocampal slice cultures was increased from 37 to 41°C for 6 h. Subsequent to this heat-shock, we investigated its impact on the morphology of the dentate gcl, on the survival of granule cells and CR cells, and on the distribution and proliferation of microglial cells in the dg.

## Materials and Methods

### Animals

Wistar rat pups (postnatal day 5) were used for hippocampal slice cultures. Animals were bred and maintained in accordance with the animal care guidelines of the institutional guidelines of the University of Bochum. All animals were housed at 22°C on a 12 h light/dark cycle with *ad libitum* food and water access. Experiments were performed in accordance with the German law on the use of laboratory animals.

### Organotypic Slice Cultures and Heat-Shock

Rat hippocampal slice cultures were used for heat-shock studies. All solutions used for organotypic slice culture preparation were sterile, and all preparations were performed in a laminar air flow bench with horizontal counter flow (Heraeus Instruments, Hanau, Germany). Five-days-old wistar rats (P5) were decapitated, and the hippocampus was dissected out and gently placed on the platform of a McIlwain tissue chopper. The hippocampus was cut perpendicular to the longitudinal axis into 400 μm thick slices, which were transferred into incubation solution at 4°C (Gey’s Balanced Salt Solution containing 10% Kynurenine and 45% D-glucose). After 1 h of recovery, the slices were transferred to a separate membrane insert (Millicell 0.4 μm culture plate inserts, 30 mm diameter; Merck Millipore) and subjected to a different experimental condition (i.e., one slice of each pair served as a control, whereas the corresponding slices were subjected to experimental treatment). The cultures were incubated *in vitro* in a 37°C, 5% CO_2_ humidified incubator; 100 ml culture medium consisted of 50% MEM, 25% Hank’s balanced salt solution, and 25% heat-inactivated horse serum, supplemented with 1% glutamine (200 mM), 1.56% glucose (45% in aqua dest.), 0.58% NaHCO_3_ (7.5%, HyClone GE Healthcare), 1% penicillin, and streptomycin (Thermo Fisher Scientific/Invitrogen). To simulate a fever situation *in vitro*, heat-shock of the cultures started at DIV 3. Thus, the incubation temperature for the hippocampal slice cultures was increased to 41°C for 6 h to achieve hyperthermia. Next, the slice cultures were again incubated at 37°C for 2 more days. Slices were subsequently fixed with 4% paraformaldehyde (PFA) in phosphate buffer (PBS) for 90 min. After that, the hippocampal sections were washed and transferred in PBS. The outcome was compared with the control slice cultures exposed all days at 37°C, 5% CO_2_.

### Immunohistochemistry and Image Capture

The collected slice cultures in PBS were subsequently processed free-floating and all incubations were performed darkened on a shaker. For immunostaining, tissue sections were permeabilized in 0.1% Triton-X-100 in PBS for 1 h at room temperature and then pre-incubated with blocking solution (5% normal goat serum, 1% BSA in 0.1 M PBS, 0.1% Triton-X-100) overnight at 4°C. After washing 3x in 0.1 M PBS for 10 min, sections were incubated in primary antibodies diluted in 0.1 M PBS for two nights at 4°C. The following primary antibodies were used: rabbit polyclonal anti-Iba-1 (1:500; Wako Pure Chemical Industries, Osaka, Japan) or mouse monoclonal anti-ED-1 (1:250; Abcam^®^, Cambridge, United Kingdom). After primary antibody incubation, sections were washed twice in 0.1% Tween-20 in PBS and once in PBS for 5 min, before secondary antibodies were applied for 3 h at room temperature: Alexa Fluor 594-coupled goat anti-rabbit IgGs (1:400; Invitrogen, Frankfurt, Germany) or Alexa Fluor 488-coupled goat anti-rabbit IgGs (1:400; Invitrogen). Sections were washed again and treated with the primary antibodies diluted in 0.1 M PBS overnight at 4°C: anti-Reelin (1:1000; Medical & Biological Laboratories Co., Tokyo, Japan) or anti-Prox-1 (1:1000; AngioBio Co., San Diego, CA, United States). After washing, they were incubated with secondary antibodies conjugated with Alexa 488 or 594 fluorophores (1:400) for 2 h at room temperature. Nuclei were counterstained with the fluorescent dye 4′,6-diamidino-2-phenylindole (DAPI) (1:1000; Roche Diagnostics GmbH, Mannheim, Germany). Finally, after three repetitive washing with PBS, free-floating sections were mounted on glass slides. All sections were embedded with fluorescent mounting medium (Carl Roth GmbH & Co. Kg, Karlsruhe, Germany), cover-slipped, and subsequently viewed and photographed using confocal fluorescence microscope (Nikon Spinning Disk). For image acquisition and quantification, a 20x air- and 60x water-magnification immersion objective was used. The sections were also processed by using the ImageJ 1.52k software. To control for specificity, sections were processed according to the protocol above with primary antibodies omitted.

### Measurement of Granule Cell Layer Width

**FIGURE 1:**
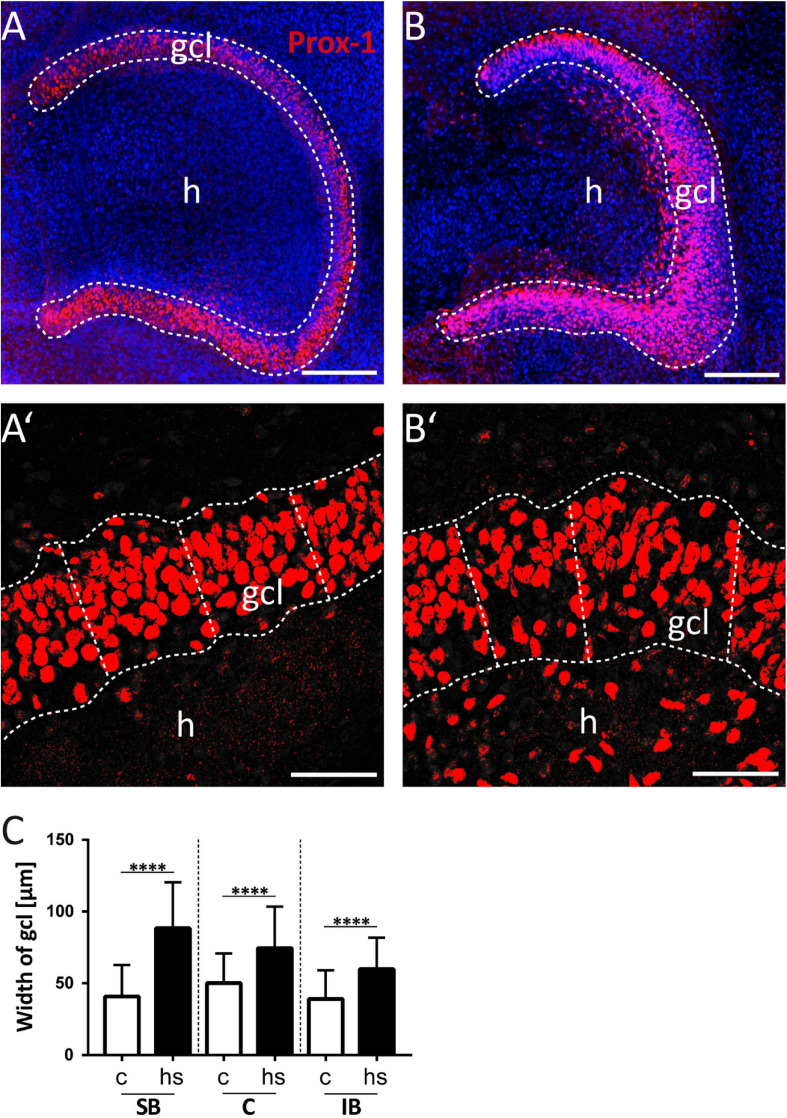
Heat-shock increases the width of the granule cell layer (gcl). **(A)** Prox-1 immunopositive staining under control condition and **(B)** after heat-shock. **(A’,B’)** The width of the granule cell layer was determined by measuring the shortest distance from Prox-1 immunostained (red) granule cell nuclei at the inner (hilar) border of the gcl to the most distal granule cell nuclei neighboring the molecular layer. Prox-1-stained nuclei with a distance of more than 30 μm from the granule cell layer were excluded from the measurements. Slices were counterstained with the nuclear dye DAPI (blue). **(C)** The mean and the standard deviation of three regions of interest (see Figures 4A,D) in three optical z-sections were calculated for each case and showed a significant increase in width of the granule cell layer after heat-shock (hs) compared to control (c) independent of the region of interest. Asterisks indicate significance for *p* < 0.05. gcl, granule cell layer; h, hilus. Scale bar **(A,B)**: 200 μm; **(A’,B’)**: 50 μm; *n* = 9.

**FIGURE 2:**
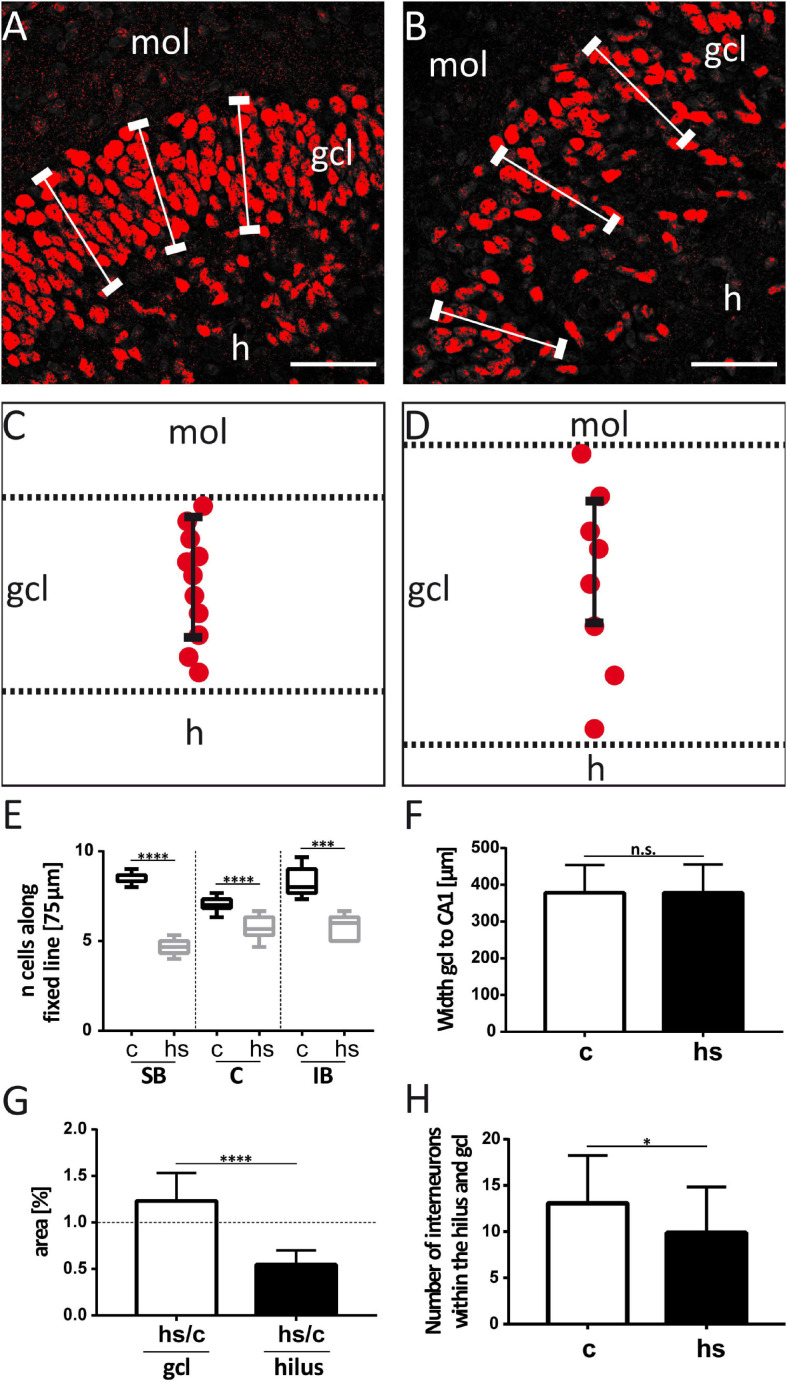
Analysis of heat-shock-induced granule cell dispersion. For counting of Prox-1 immunostained granule cells (red), standard rulers (fixed lines) of 75 μm length were positioned into confocal images of the granule cell layer of control slices **(A)** and after heat-shock **(B)**. Schematic drawings (**C**, control; **D**, heat-shock) illustrate that the cell number that is covered by such a ruler is reduced after heat-shock, indicative of granule cell dispersion. **(E)** Counting of cells covered by rulers positioned in different subregions of the granule cell layer (gcl) showed a decrease in the number of granule cells in all subregions. Boxplots with median, 25% and 75% quartile, minimum, and maximum are shown. *n* = 9. **(F)** The shortest distance from the granule cell layer to the CA1 pyramidal cell layer region was determined in a standardized manner by measuring the shortest distance of the intermediate part of the gcl suprapyramidal blade (SB) to the intermediate part of the CA1 pyramidal cell layer. This distance did not change between control- (c) and heat-shock-treated slices (hs). *n* = 9. **(G)** However, when comparing the surface area covered by gcl and hilus in control and heat-shock-treated slices, the gcl area was significantly increased, while the hilus area was significantly decreased. *n* = 9. **(H)** Cell counts revealed a slight, but significant reduction of Reelin positive interneurons after heat-shock in the area covered by the hilus and gcl. *n* = 5. Asterisks indicate significance for *p* < 0.05. mol, molecular layer; h, hilus; C, crest; IB, infrapyramidal blade. Scale bar (right lower corner): 50 μm.

**FIGURE 3:**
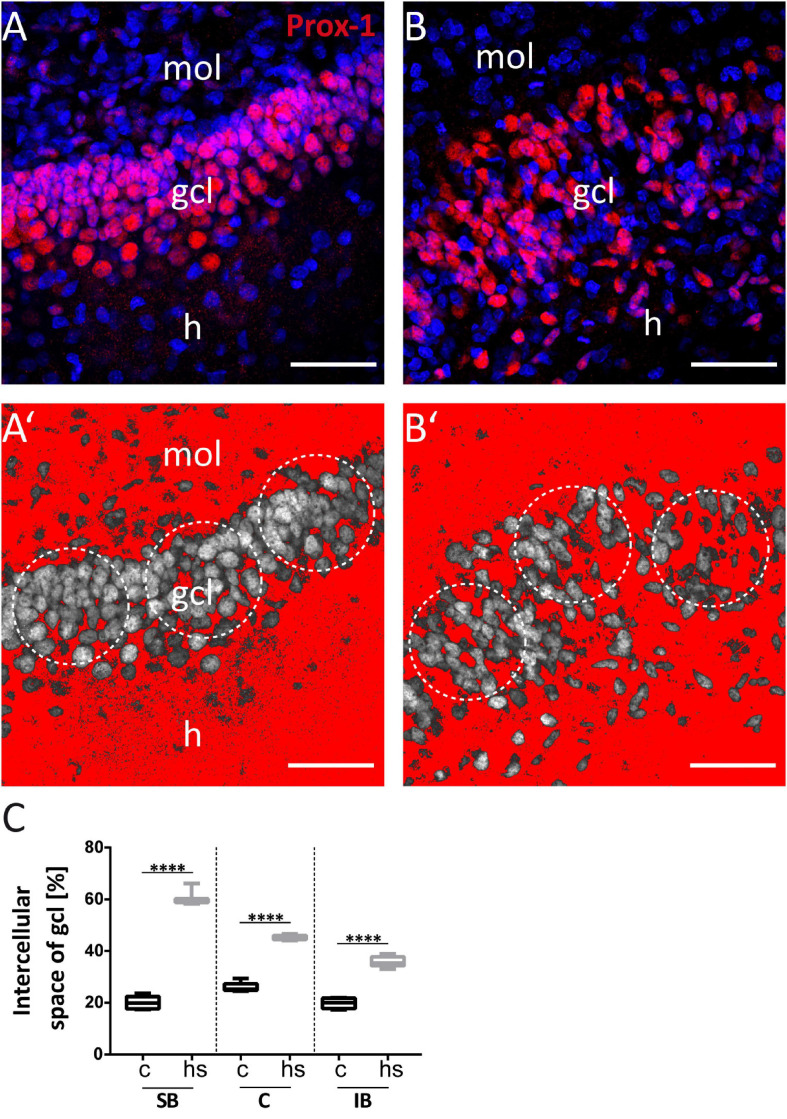
Increase of extracellular space in the granule cell layer after heat-shock. Representative Prox-1 immunostaining of granule cells (red) under control conditions **(A)** and after heat-shock **(B)**. Same images are shown with a threshold dependent mask (red) around granule cells (gray) (**A’**, control; **B’**, heat-shock). Circular masks (white dotted circles) were used to measure the approximate area increase between cells after heat-shock compared to control **(C)**. Boxplots with median, 25% and 75% quartile, minimum, and maximum. *n* = 9. Asterisks indicate significance when *p* < 0.05. gcl, granule cell layer; mol, molecular layer; h, hilus. Scale bar: 50 μm.

To evaluate a potential GCD in the dg, three different approaches with ImageJ were used. The average width of the gcl was measured by length measurement (1) according to Houser (1990), by counting the number of cells covering a 75 μm standard ruler (2), and by evaluation of the area of intercellular space in the gcl (3). The average width of the gcl of the dg was determined in DAPI- and Prox-1-stained sections of heat-shock-treated and control hippocampi in each subregion. (1) The perpendicular distances from the inner (hilar) border of the gcl to the outer border of the most distal granule cell somata were determined by DAPI and Prox-1 immunostaining and using ImageJ (Figure 1). The mean and SD of three regions of interest in three optical z-sections were calculated for each case. (2) For more precise analysis, a fixed line (ruler) with a defined length (75 μm) was placed perpendicularly to the gcl over each subfield at defined intervals (Figure 2). To quantify the width of the gcl, only granule cells in proximity to or in contact with the standard ruler were chosen. Cells were excluded from quantification when the cell body was not positioned on the fixed line. (3) The intercellular space within the gcl was calculated automatically in a threshold dependent manner by using ImageJ. Thereby, the intercellular space of the gcl was compared between matched slices of the same animal, i.e. between control and after heat shock. In each of the three positions [suprapyramidal blade (SB), crest (C), and infrapyramidal blade (IB)], a fixed region of interests (circle) was placed three times per region in the gcl and intercellular space was calculated (illustrated in Figure 3). To find out whether the heat-shock caused a generalized tissue expansion of the hippocampal slice culture, we measured the shortest distance between the SB of the gcl and the CA1 pyramidal cell layer in a standardized manner. Next, we compared the area of the gcl and hilar region in control and heat-shock-treated slice cultures by measuring the pixel area size with standard implemented features in ImageJ and normalized the area size after heat-shock to the area size of the matched control slice.

### Cell Quantification

**FIGURE 4:**
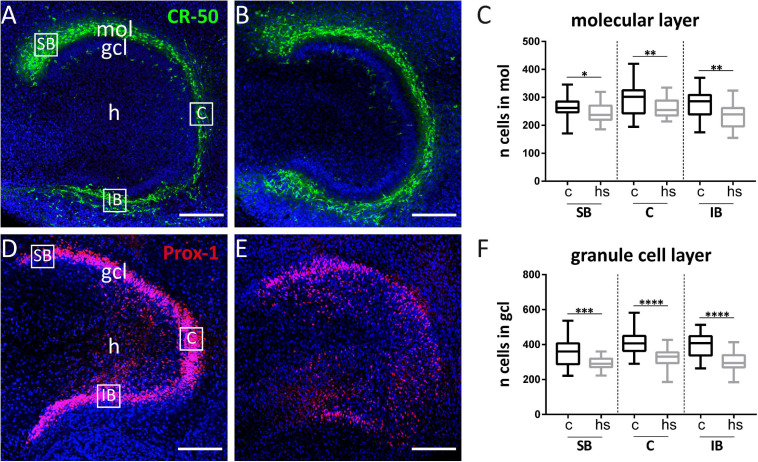
Effect of heat-shock on cell viability in hippocampal slice cultures. **(A)** Representative immunofluorescent staining against Reelin (green) of control hippocampal slice culture. Cajal–Retzius (CR) cells in the molecular layer (mol) of the dentate gyrus appear green. White squares enclose three subregions of interest within the mol, which were chosen for cell counting (same, enlarged areas of interest are shown in Figure 5). **(B)** CR-50 staining against Reelin (green) of a heat-shock-treated hippocampal slice culture. **(C)** Quantification of DAPI positive cells in the dentate molecular layer in control slice cultures (c) and after heat-shock (hs). **(D)** Prox-1 immunostaining visualizing granule cells (red) in a control hippocampal slice culture. **(E)** Prox-1 immunostaining in dentate gyrus after heat-shock. Note that the compactness of the granule cell layer (gcl) is lost. Nuclei are counterstained with DAPI (blue) in all images. Quantification of CR-cells within regions of interest (SB = suprapyramidal blade, C = crest region, and IB = infrapyramidal blade) is shown in **(A)**, squares were positioned on the gcl for granule cell counts and quantified in the same way **(D)**. After heat-shock, the total cell number decreases significantly independent of its position in mol or gcl, although a stronger reduction is seen in the gcl **(F)** compared to the mol **(C)**. Boxplots show median, 25% and 75% quartile, minimum, and maximum. *n* = 27. Asterisks indicate significance for *p* < 0.05. gcl, granule cell layer; h, hilus. Scale bar: 200 μm.

For quantitative analysis of cell distribution in hippocampal slice cultures, identical illumination for each section by a photographer who was blinded to the experimental condition was used. High-resolution images acquired with a 60x water immersion objective (Nikon 60xA/1.20) within subfields of the dg (SB, C, and IB; white boxes indicate the areas of interest in Figures 4A,D) in particular of the molecular (mol) and the gcl were taken for visual cell counts. For further analysis (also blinded), two pictures per hippocampal subregions in three z-levels were chosen.

The total number of cells was counted visually in optical sections by staining with DAPI. Individual cell counts were achieved by staining with cell specific markers. Thus, quantitative analysis of different cell types in the dg subfields (SB, C, and IB) was perfomed by counting cells that were immunopositive for CR-50 (CR-cells), Prox-1 (granule cells), Iba-1 or ED-1 (microglia), respectively. Cell numbers were normalized to the total number of DAPI stained cells per image subfield.

Reelin positive interneurons were manually counted in optical sections from matched slices of five different animals per experimental condition.

Propidium iodide (PI) staining was performed by applying 200 μl/ml of a 500 nM PI solution to living cell cultures 1 h before fixation with PFA. Slice cultures were then co-stained with Prox-1 as described earlier. Cell counts of PI positive cells in the gcl were achieved visually by an experimenter blinded to the experimental condition in one exemplary z-level of a slice.

### Statistical Analysis

Statistical analyses were performed with GraphPad Prism version 7.00 (GraphPad Software Inc., San Diego, CA, United States) and in Microsoft Excel. For analysis, slices from the same animal incubated as control or treated as heat-shock were tested as pairs of groups. These pairs of groups were tested for normal distribution (D’Agostino test and Shapiro–Wilk test) and if so a paired Student’s *t*-test was performed. In the case of non-normal distribution of the raw data, the Wilcoxon–Mann–Whitney test was used for statistics. Values were considered statistically significant at *p* < 0.05. Statistics reported in the text and figures are represented either by mean value and standard deviation or by boxplots giving the median (middle dash), the lower and upper quartile (box borders), and the minimum and maximum values (whiskers).

## Results

### Heat-Shock Induces Granule Cell Dispersion in Hippocampal Slice Cultures

To study the impact of a heat-shock on the morphology of the dg, we first analyzed the width of the gcl in matched hippocampal slice cultures after heat-shock and in untreated control cultures (Figures 1A,B) by measuring the perpendicular distance from the inner (hilar) edge of the gcl to the most distal granule cell somata bordering the dentate molecular layer (Figures 1A’,B’) (Houser, 1990). This measurement revealed a highly significant increase in width of the gcl after heat-shock, of 47.6 μm for the SB region, of 24.4 μm for the C region, and of 20.9 μm for the IB region (Figure 1C, c = control, hs = heat-shock). Next, we used two different approaches to quantify this change of width of the gcl (Figures 2, 3). Thus, we first counted the number of granule cells that a ruler of 75 μm length encompassed in the gcl under control condition (Figure 2A) and after heat-shock (Figure 2B). Hyperthermia significantly reduced the number of granule cells that were encompassed by the ruler in all analyzed subregions (SB, C, and IB) of the dentate gcl (Figure 2E). This result is schematically illustrated in Figures 2C,D, indicating a reduction of the cell number in the gcl and an increase in width. Note that ruler encompassed the entire width of the gcl in control slices but not after heat-shock (compare Figures 2A,B and C,D). The distance between the gcl and CA1 pyramidal cell layer showed no difference between matched slices of control and heat-shock-treated cultures (Figure 2F). However, when measuring the gcl area of heat-shock-treated cultures normalized to the gcl area of untreated controls from matched slices, we found the area of the gcl increased by 23% whereas the area of the hilus in the same slice decreased by 45% (Figure 2G). Counting of Reelin expressing hilar interneurons revealed that the number of interneurons was slightly, but significantly reduced after heat-shock (Figure 2H). Next, we analyzed the apparent expansion of the extracellular space between granule cells after heat-shock. For quantification, we positioned a circular mask into the dentate gcl, calculated the extracellular space between the cells within the circle in an automatized threshold-dependent manner (Figures 3A’,B’), and normalized to the total area of the circular mask. Figure 3 shows Prox-1 positive staining of untreated granule cells (Figure 3A) and of granule cells after heat-shock (Figure 3B) in the SB of the gcl. Comparison of these images visualizes the reduced cell packaging in the gcl after heat-shock. This revealed a significant increase in extracellular space after heat-shock treatment (Figure 3C).

### Survival of Dentate Granule Cells but Not of CR Cells Is Impaired After Heat-Shock

**FIGURE 5:**
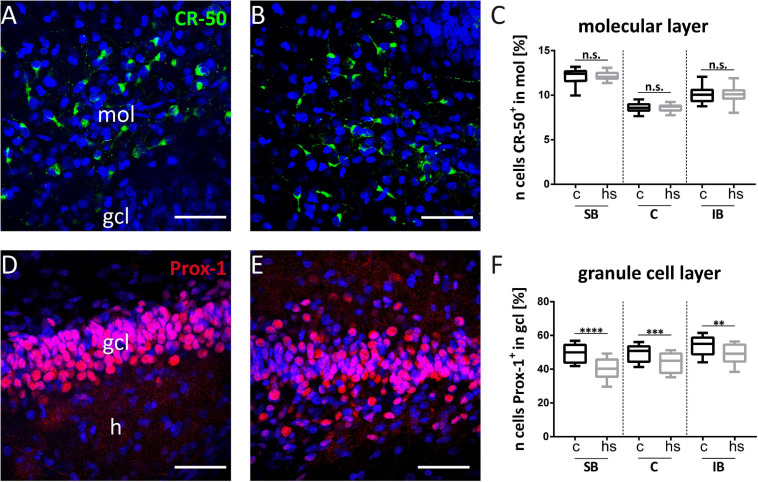
Heat-shock reduces the number of granule cells but not of CR cells. Exemplary, high magnifications of CR (green) cells immunostained against Reelin are shown within the dentate suprapyramidal blade under control condition **(A)** and after heat-shock **(B)**. Prox-1 immunostained granule cells (red) are shown in a control slice **(D)** and after heat-shock **(E)**. Counts of CR cells within the molecular layer (mol) show comparable cell numbers **(C)**, whereas granule cell counts revealed a significant reduction in all subregions of the dentate granule cell layer (gcl) investigated **(F)**. Boxplots with median, 25% and 75% quartile, minimum, and maximum. *n* = 27. Asterisks indicate significance for *p* < 0.05. gcl, granule cell layer; h, hilus. Scale bar: 50 μm.

**FIGURE 6:**
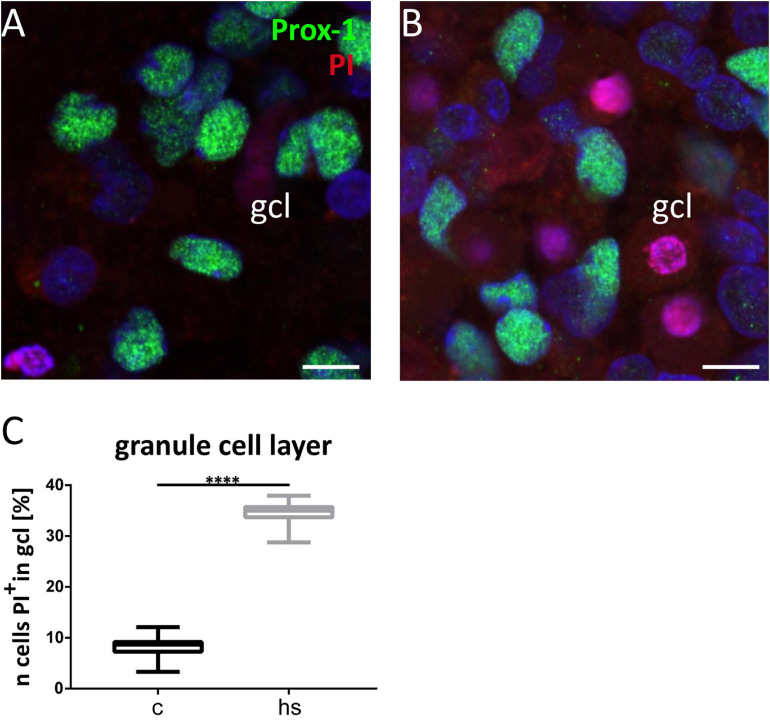
Increased cell mortality in the granule cell layer (gcl). Detail of the gcl visualized by Prox-1 immunostaining of granule cells (green) under control conditions **(A)** and after heat-shock **(B)**, combined with propidium iodide (PI) staining of dead cells with pyknotic nuclei (red). Quantification of PI positive cells in gcl **(C)** revealed a significant increase after heat-shock. Boxplots with median, 25% and 75% quartile, minimum, and maximum. *n* = 30. Asterisks indicate significance for *p* < 0.05. gcl, granule cell layer. Scale bar: 10 μm.

To analyze a potentially deleterious effect of the heat-shock on survival of dentate granule cells, we determined the total cell number in confocal images of the gcl and mol in hippocampal slice cultures in control slices and after heat-shock (Figure 4) by counting DAPI positive nuclei. These counts revealed a highly significant reduction of the cell number in the mol (Figure 4C) and gcl after heat-shock (Figure 4F). To identify individual cell types, slices were immunostained with a Reelin-specific antibody to label CR cells (Figures 4A,B) and with an antibody against Prox-1 (Prospero homeobox protein 1) to label dentate granule cells (Figures 4D,E). High-resolution confocal scanning of different representative areas within the dg was performed (Figures 4A,D; SB, C, and IB) and immunostaining against Reelin and Prox-1 allowed for counting of individual cell types in the dg of control (Figures 5A,D) and after heat-shock (Figures 5B,E). Surprisingly, cell counts for Reelin positive CR cells within three representative areas of the dentate molecular layer (SB, C, and IB) showed no significant difference after heat-shock (Figure 5C), whereas the number of Prox-1 positive granule cells was significantly reduced (Figure 5F). Next, we quantified dying cells by counting the number of PI positive pyknotic nuclei of cells in the gcl after heat-shock (Figure 6). In control slices, only few cell nuclei were found to be PI positive (Figure 6A, red) surrounded by numerous Prox-1 positive cells (Figure 6A, green). In contrast, after heat-shock, many PI positive cell nuclei appeared (Figure 6B, red) in the gcl and quantification confirmed that this increase was significant (Figure 6C).

### Microglia Response to Heat-Shock in the Dentate Gyrus of Slice Cultures

**FIGURE 7:**
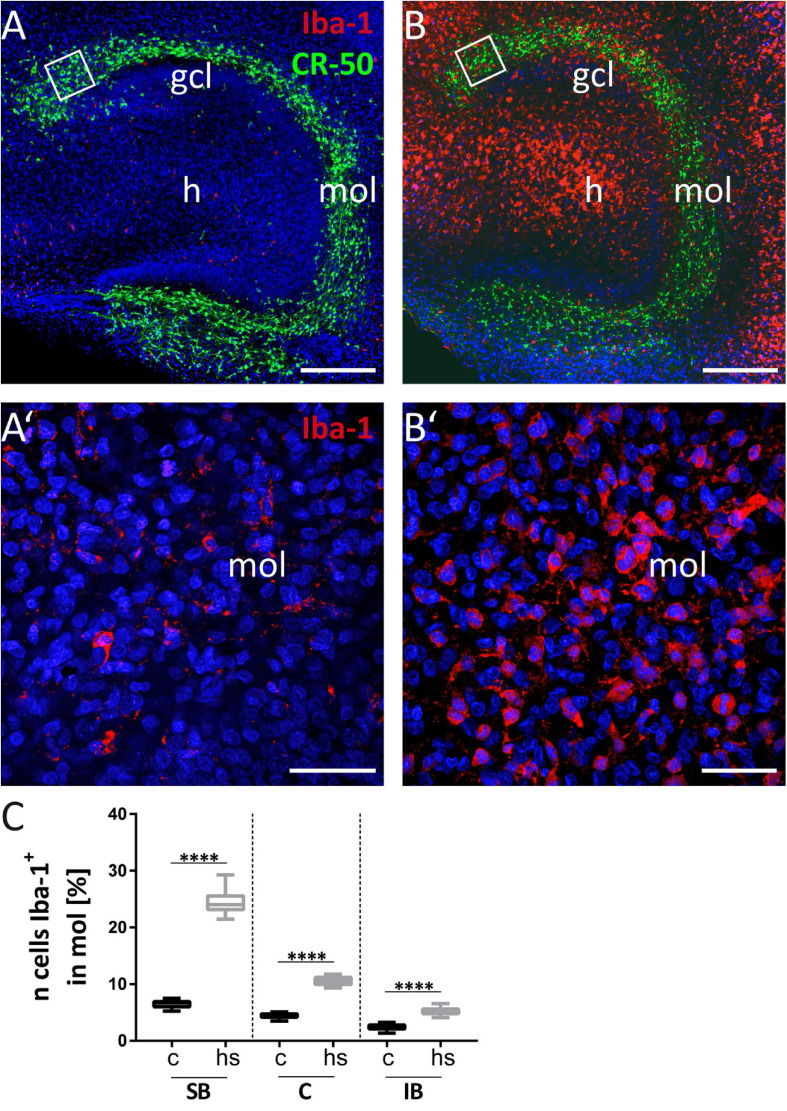
Microgliosis in dentate molecular layer (mol). **(A)** Reelin immunostaining to visualize CR cells (green) combined with Iba-1 staining (red) to stain microglia. White squares in **(A)** and **(B)** are enlarged in **(A’)** and **(B’)** at higher magnification. **(B)** After heat-shock strong Iba-1 positive immunostaining (red) is seen particularly in the hilus (h), but also in the gcl and mol. **(A’)** Only few Iba-1 positive microglial cells (red) are seen in the mol in control slices. **(B’)** Numerous microglial cells are detectable by Iba-1 staining (red) in the mol after heat-shock. **(C)** Cell counts in boxed areas of the dentate mol show a significant increase of microglia cells in all subregions investigated. Boxplots are shown with median, 25% and 75% quartile, minimum, and maximum. *n* = 27. Asterisks indicate significance when *p* < 0.05. gcl, granule cell layer; mol, molecular layer; h, hilus. Scale bar **(A,B)**: 200 μm; **(A’,B’)**: 50 μm.

**FIGURE 8:**
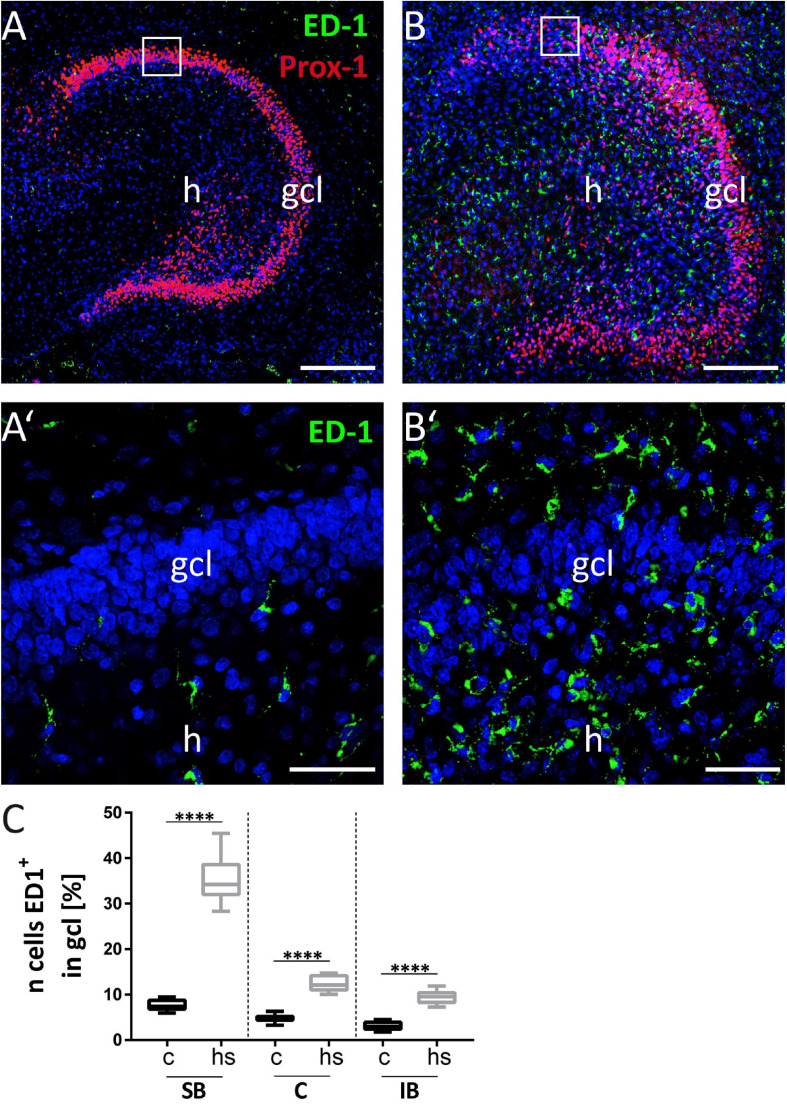
Increased microgliosis in dentate granule cell layer (gcl). **(A)** Prox-1 immunostaining to label granule cells (red) in combination with anti-CD68 immunostaining (ED-1; green) to label microglia shows weak immunopositive signals for CD68 protein in control (boxed area of the gcl in **A**, enlarged in **A’**). **(B’)** After heat-shock, a strong immunopositive signal for the microglia marker ED-1 (green) can be seen. Enlargement of area is indicated by white square in **(B)**. **(C)** Quantification of microglia cell numbers in gcl after heat-shock shows a significant increase, even more prominent when compared to the microglia increase in the mol (also compared to Figure 7C). Boxplots are shown with median, 25% and 75% quartile, minimum, and maximum. *n* = 27. Asterisks indicate significance when *p* < 0.05. gcl, granule cell layer; h, hilus. Scale bar **(A,B)**: 200 μm; **(A’,B’)**: 50 μm.

Microglial cells can actively migrate to lesion sites with ameliorating effects supporting recovery after lesion (Kettenmann et al., 2011; Sieger et al., 2012; Eyo and Dailey, 2013). The ionized calcium-binding adapter molecule 1 (Iba-1) is a commonly used marker for microglia. Combination of the antibodies Iba-1 and CR-50 against Reelin allowed to analyze the impact of the microglia response to heat-shock in the dg. Figure 7A shows that under control conditions, microglial staining with an antibody against Iba-1 is sparse in both the gcl and the mol (enlargement of mol shown in Figure 7A’, indicated by the boxed area in Figure 7A). After heat-shock, the slice cultures show a strong increase of Iba-1 positive staining in the mol (Figure 7B, enlargement in B’). Microgliosis is also evident in the hilar region and pyramidal cell layer of the hippocampus (Figure 7B). Counting of microglia cells based on Iba-1 positive staining show a significant increase in microglia by more than twofold in all three analyzed dg subregions (Figure 7C). To count microglia in the gcl before treatment (Figures 8A,A’) and after heat-shock (Figures 8B,B’), the ED-1-antibody against the cluster of differentiation protein (CD68) was used to label microglia in combination with a Prox-1 antibody to visualize granule cells. As already shown in the previous experiment, only few microglia cells were detected in hippocampal sections under control conditions, particularly in the gcl (Figure 8A, white square in Figure 8A depicts the boxed area in Figure 8A’). Only few ED-1 positive cells were detected at the inner border of the gcl to the hilar region (Figure 8A’). This changes significantly after heat-shock, as can be seen in Figure 8B (and in magnification in Figure 8B’), showing an increased intensity of immunostaining against CD68 protein (ED-1, green), indicative of massive microglia invasion into the gcl and adjacent areas. Quantification of microglia cells in this cell layer also showed a significant increase more than threefold in all three investigated subregions of the dg (Figure 8C).

**FIGURE 9:**
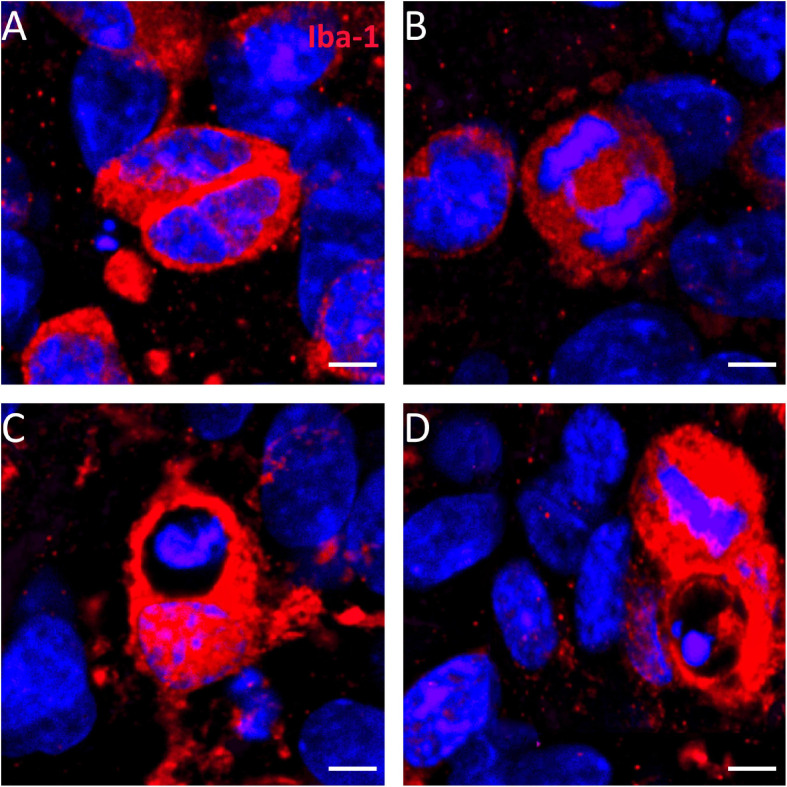
Mitosis contributes to increased number of microglia in the molecular cell layer. When combining Iba-1 staining (red) with DAPI nuclear staining (blue), we found diverse cell division stage of microglia in hippocampal slice cultures after heat-shock **(A,B)** but not in control slices (not shown). **(C,D)** Numerous microglia that phagocytize cell debris can be seen after heat-shock in hippocampal slice cultures. Scale bar: 10 μm.

High-magnification images of heat-shock-treated microglia stained against the Iba-1 protein demonstrate that the increased microglia staining resulted, at least in part, from mitotic cell divisions (Figures 9A,B). Numerous dividing cells were found in the dg of heat-shock-treated slice cultures. In addition, numerous actively phagocytizing microglia cells were found within heat-shock-treated hippocampal slice cultures (Figures 9C,D).

## Discussion

Ammonshorn sclerosis and/or dispersion of dentate granule cell are frequently observed in epileptic hippocampal tissue, and febrile seizures early in life are suspected to be causally linked to these pathologies. Loss of Reelin, a protein known to control neuronal migration during development, has been discussed as a possible cause of pathological GCD. In turn, recent findings suggest that GCD may not always be disease related but could instead reflect normal morphological variation. For instance, the presence of newborn granule cells within the dentate hilar region during early dg development reflects the normal development of this region but could be misinterpreted as pathological GCD. Thus, the interpretation of GCD in a disease-related context remains controversial. To explore the potential impact of febrile temperature on granule cells and Reelin secreting CR cells, hippocampal slice cultures were subjected to a transient temperature increase (heat-shock). We found that a heat-shock caused both dentate GCD and degeneration of a fraction of granule cells. Surprisingly, the viability of CR cells was not affected. This precludes that heat-shock-induced GCD was related to depletion of the CR cell population. We also found that the heat-shock caused a massive increase in the number of microglial cells in the dg, indicating a robust immune response to the heat-shock-induced tissue damage.

### Granule Cell Dispersion in Development and Disease

A developmental peculiarity that distinguishes dg (archicortical) development from neocortical development is the emergence of three consecutive germline matrices during dg development. In the tertiary proliferation zone located in the hilar region of the early postnatal dg, proliferating progenitors generate granule cells which migrate from the hilar region to their final positions in the compact gcl (Altman and Das, 1965; Sugiyama et al., 2013; Hayashi et al., 2015). Thus, the transient distribution of newborn granule cells in the hilar region is a normal stage of early postnatal dg development but could be easily mistaken as pathological GCD. Also in the reeler mutant mouse which does not express the extracellular matrix protein Reelin, dentate granule cells are formed in the tertiary proliferation zone, but fail to migrate properly to form a compact gcl and therefore remain dispersed in the dentate hilar region throughout life (Stanfield and Cowan, 1979; D’Arcangelo et al., 1997; Forster et al., 2006a). GCD has also been observed in resected hippocampal tissue of patients with TLE, often associated with ammonshorn sclerosis (Houser, 1999; Haas and Frotscher, 2010). Since an inverse correlation was found between the number of Reelin-expressing cells in the hippocampus and the extent of GCD in TLE patients, a causal relationship between reduced Reelin availability and GCD had been suggested (Haas et al., 2002; Haas and Frotscher, 2010; Bozzi et al., 2012). In turn, a recent study reported on GCD in the hippocampus of pediatric patients that did not suffer from epilepsy, indicating that GCD, at least at early age, might be within the normal variation range of dg morphology (Roy et al., 2020). Thus, careful interpretation of the causes that might underlie GCD should take into account that morphological variation or apparent dispersion at early stages might reflect newborn granule cells that migrate from the tertiary proliferation zone to form a compact gcl. Since defined transcription factors, including for example Pax-6 and Prox-1, are successively expressed in granule cells and their precursors during dg development (Sugiyama et al., 2013), analysis of transcription factor expression may be useful to distinguish pathological from normal developmental GCD. Characteristic pathological GCD that is clearly not related to development, but pathology-related, has been described in rodents *in vivo* after administration of the epilepsy inducing drugs pilocarpine (Gong et al., 2007; Curia et al., 2008) or kainic acid (Haas et al., 2002; Heinrich et al., 2006; Antonucci et al., 2009; Duveau et al., 2011).

### Heat-Shock-Induced Granule Cell Dispersion in Hippocampal Slice Cultures

We wondered whether a transient temperature increase might be sufficient to induce GCD *in vitro*, and therefore we subjected hippocampal slice cultures from young postnatal rats to a heat-shock of 41°C for 6 h. Our experiments revealed that this transient heat-shock is sufficient to induce GCD in hippocampal slice cultures. What might be the underlying causes that induce GCD in our *in vitro* model? For instance, a reduction in the number of Reelin-secreting CR cells, implying reduced Reelin availability, had earlier been shown to be a potential trigger for GCD (Haas et al., 2002; Haas and Frotscher, 2010). Along this line, also kainate induced loss of Reelin expressing hilar interneurons was found to correlate with GCD, suggesting that loss of interneuron-derived Reelin might trigger GCD (Orcinha et al., 2016). Thus, reduced Reelin availability might destabilize the compact gcl and cause displacement of differentiated granule cells toward the hilar region or might fail to prevent overmigration into the dentate molecular layer, which normally does not contain granule cells (Zhao et al., 2004; Hack et al., 2007; Chai et al., 2009; Folsom and Fatemi, 2013). However, in the present study, we were surprised that the size of the CR cell population was unchanged after the heat-shock. Thus, reduced availability of CR cell-derived Reelin is an unlikely cause for heat-shock-induced GCD, though so far we cannot exclude that proteolytic processing of Reelin, which is required for proper Reelin function (Lambert de Rouvroit et al., 1999; Kohno et al., 2009; Haas and Frotscher, 2010; Tinnes et al., 2013), might be affected by the heat-shock. Also reduced Reelin availability due to a decreased number of Reelin expressing hilar interneurons caused by kainate treatment has been hypothesized to be causally linked to GCD (Heinrich et al., 2006; Haas and Frotscher, 2010; Duveau et al., 2011). In our experiments we also found a slight but significant reduction in the number of Reelin expressing hilar interneurons after heat-shock. However, selective inactivation of Reelin in interneurons of conditional Reelin knock-out mice did not destabilize the gcl (Pahle et al., 2020), arguing against a role of interneuron-derived Reelin in maintaining gcl integrity. Therefore, also other causes that might trigger GCD should be considered. Thus, careful analysis of slice cultures after heat-shock revealed that not only displacement of granule cells, as evidenced by the broadening of the gcl, but also degenerated granule cells, interspersed between the surviving granule cells, contribute to the reduced cell packaging density and to an enlargement of the extracellular space in the gcl. In fact, granule cell loss-induced tissue shrinkage and straining has been hypothesized earlier to be involved in the origin of GCD (Lurton et al., 1997). This indicates that also reorganization of the extracellular matrix may be one of the factors that contribute to pathological dispersion of granule cells. In turn, we can exclude a generalized expansion of the hippocampal slice culture tissue after heat-shock since the distance between gcl and CA1 pyramidal cell layer did not change after heat-shock.

### Granule Cell Dispersion and Seizures

An important question that is difficult to answer is whether GCD contributes to the development of seizures or rather has an anti-epileptic effect. Earlier studies suggested that individuals with GCD have an increased leakage of potassium ions from granule cells which reduces their excitability, pointing to an anti-epileptic effect (Stegen et al., 2009). In turn, mispositioning of granule cells may favor the development of epileptic discharges (Koyama, 2013). On the other hand, recent retrospective studies on hippocampi of pediatric patients with or without seizure history did not show significant differences of cell loss or GCD (Harding and Thom, 2001; Roy et al., 2020). In addition, abnormal neuronal stratification and microgliosis were also observed in the hippocampus of seizure-free individuals, suggesting that GCD cannot be considered to be pathognomonic for TLE (Roy et al., 2020), much in contrast to earlier studies which had judged GCD to be a specific histopathological feature of TLE (Lurton et al., 1997; Koyama et al., 2012; Thom, 2014). Febrile seizures and ectopic granule cell positioning were hyperthermia-induced in rats *in vivo* (Koyama et al., 2012). To analyze the mechanisms that might underly aberrant granule cell migration in this model, organotypic slice cultures were prepared from animals subjected to hyperthermia, and the authors found that aberrant granule cell migration was caused by excitatory GABAergic signaling in dentate granule cells (Koyama et al., 2012). Reeler mutant mice which display severe cortical migration defects including dentate GCD do not show spontaneous seizure activity, though precisely timed activation of hippocampal neurons is affected in reeler (Kowalski et al., 2010). On the other side, autosomal dominant mutations in the human Reelin gene were identified that are causally linked to epileptic seizures (Dazzo et al., 2015). In this context, it is also interesting to note that in human patients with complex febrile seizures in early childhood, TLE and ammonshorn sclerosis were reported to be associated with an increased number of CR cells in the hippocampus formation (Blumcke et al., 1999). Certainly, further studies are required to characterize the cascade of events that lead to dispersion of granule cells after heat-shock. While in the extracellular matrix altered availability of signaling molecules and their proteolytic cleavage may be involved, changes of the dynamics of cytoskeletal elements within the cell, such as actin filaments and microtubules, are a prerequisite for active displacement of neurons, including Reelin-controlled neuronal migration (Chai et al., 2009; Meseke et al., 2013; Forster, 2014).

### Heat-Shock Induces Massive Microgliosis in Hippocampal Slice Cultures

Microglia cells, descendants of the monocyte-macrophage lineage, are involved in the detection of cerebral dysfunctions, infections or mechanical injuries and actively migrate to pathological lesion (Sieger et al., 2012). Microglial cells are also involved in the phagocytosis of cells, excreted cell components, and misfolded proteins (Walter and Neumann, 2009; Kettenmann et al., 2011). The activation of microglia is a characteristic feature of inflammatory conditions (Vezzani and Ruegg, 2011). In addition, glial cells also participate in epilepsy-related events (Boer et al., 2006; Ravizza et al., 2006). There is recent evidence that active microglial brain surveillance prevents neuronal network hyperexcitability, while sustained reduction of microglial dynamics induces spontaneous seizures (Merlini et al., 2021). In this context, microglia may influence the heat-shock-induced tissue alterations described in the present hippocampal *in vitro* model. The increased activation of microglia is one of the main components under pathological conditions, for instance, reflecting inflammatory changes in nerve tissue (Vezzani and Ruegg, 2011). Ammonshorn sclerosis, i.e., degeneration of hippocampal neurons, is often associated with TLE (Blumcke et al., 2002) and accompanied by an increasing activation of microglial cells. Microglia may act inflammatory cytotoxic, but also beneficial neuroprotective. Thus, immunosuppressive effects on the surrounding tissue and cells were described (Colton, 2009; Kigerl et al., 2009; Czeh et al., 2011; Kettenmann et al., 2011; Tang and Le, 2016), and the pathological course of damaged tissue might be influenced in a positive way by invasion of microglia.

In our heat-shock model, we observed that the heat-shock-induced tissue damage was followed by a massive increase in the number of microglial cells in the dg, particularly in the gcl. Our findings suggest that subregion- and cell layer-specific differences exist in the microglial response to heat-shock (see Figures 7, 8), likely reflecting local differences in inflammatory reactions. Accordingly, the gcl, where a large number of neurons were damaged by the heat-shock, was infiltrated by microglia cells. Microgliosis was at least in part caused by an increased mitotic cell division of microglia, as witnessed by microglia mitotic figures in the slice cultures after heat-shock (see Figures 9A,B). In addition, infiltration of migrating microglia contributed to microgliosis. However, in other studies, microglia activation was also observed without cell loss, apparently induced by seizures alone, and without immediate synthesis of inflammatory mediators (Vezzani et al., 2000; Ravizza et al., 2008; Dube et al., 2010; Maroso et al., 2011; Devinsky et al., 2013). To answer the question to what extent the heat-shock itself or liberation of inflammatory mediators contributes to the activation of microglia, further experimental studies are required. If phagocytosis of dead cells reduces the damage of the hippocampus (see Figures 9C,D), their elimination would be an essential component in the remission of pathological changes after heat-shock. Further studies are required to assess a positive curative effect or, in the case of incomplete phagocytosis, a detrimental effect on the pathogenesis. Thus, potentially microglia activation could represent a measurable parameter of the immune system attempting to reduce neuronal cell loss. For instance, it cannot be conclusively determined whether the increase in the number of activated phagocytic microglial cells [rounded phagocytic type (RPT)] indicates a pro- or an anti-inflammatory potential or a pro- or an anti-convulsive effect, respectively. Of course, our results cannot be directly transferred to the interpretation of epilepsy-affected hippocampi, because the slice cultures were derived from healthy rats not suffering from epilepsy. They might, however, be useful as a model to interpret the role of an inflammatory state in the brain, possibly favoring the development of epilepsy. In this context, it should be noted that Roy et al. (2020), who recently reported on GCD as a non-specific finding in pediatric patients, did not observe a microglia reaction in the analyzed hippocampal tissue with no history of seizures. This finding, together with our observation of microgliosis after heat-shock, underpins the importance of microgliosis as an important parameter to distinguish pathological from non-specific granule dispersion.

Another question that remains to be addressed is whether the transient temperature increase induces epileptic activity in the hippocampal slices. Studies on hyperthermia-induced seizures in immature rats were performed earlier (Holtzman et al., 1981; Tancredi et al., 1992; Tsai and Leung, 2006), with electrocortical paroxysmal discharges reported to be similar to those in infants (Holtzman et al., 1981). Results with an altered excitability in slice cultures suggest that anti-inflammatory and protective effects of microglia may influence the outcome of the pathology (Colton, 2009; Kigerl et al., 2009; Czeh et al., 2011; Kettenmann et al., 2011; Tang and Le, 2016). In this regard, it could be interesting to explore if manipulation of the heat-shock-induced microglial response may promote neuronal survival and to reduce damage of surviving neurons. However, microglial cells could also maintain the inflammatory state in the brain or activate other inflammatory pathways, confirming the concept of an induced inflammation and enhancement of pathology by glial cells (Stoll and Jander, 1999; Vezzani et al., 2011; Galic et al., 2012; Devinsky et al., 2013). Thus, microglial inactivation could contribute to a possible improvement of the cerebral state, by reducing the amount of neurotoxic factors, even if the underlying cause of the disease may not be cured (Nelson et al., 2002). Overall, currently, few studies on effects of heat-shock on neural tissue *in vitro* exist. Here we provide evidence that microglia cells play a remarkable role in the heat-shock response in hippocampal slice cultures. The relationship between heat-shock, induced GCD and microglia activation is largely unexplored. Further experimental studies on the interplay between neuropathology and the microglia response may help to progress existing therapeutic options in the pathogenesis of febrile seizures and other neurological diseases.

## Data Availability Statement

The original contributions presented in the study are included in the article/supplementary material. Further inquiries can be directed to the corresponding author/s.

## Ethics Statement

The animal study was reviewed and approved by Landesamt für Natur, Umwelt und Verbraucherschutz Nordrhein-Westfalen.

## Author Contributions

JW and MM performed experimental work and statistical analysis of data, and contributed to data interpretation and manuscript writing. SR performed experimental work on Prox-1 in combination with PI staining. EF developed the concept of the study and was involved in data interpretation and manuscript writing. All authors contributed to the article and approved the submitted version.

## Conflict of Interest

The authors declare that the research was conducted in the absence of any commercial or financial relationships that could be construed as a potential conflict of interest.
